# Critical matriarchal futures: reimagining public health communication leadership as response to state-sanctioned violence

**DOI:** 10.3389/fpubh.2026.1831079

**Published:** 2026-06-23

**Authors:** M. L. Ponder, N. Patterson, K. Reid

**Affiliations:** 1 Department of Communication, Culture and Media Studies, Cathy Hughes School of Communications, Howard University, Washington, DC, United States; 2 Department of Public Health, School of Nursing and Health Sciences, The College of New Jersey, Ewing Township, NJ, United States; 3 Department of Nutrition and Public Health, University of Saint Joseph, West Hartford, CT, United States

**Keywords:** black feminist thought, critical matriarchy, decolonial frameworks, health communication, health equity, public health workforce, state-sanctioned violence

## Abstract

This commentary argues that the crises facing the U.S. public health workforce - marked by mass attrition, particularly among Black women, increasing incidence of disease and illness, loss of critical social safety nets, mediated surveillance, and eroding public trust - represent a manifestation of state-sanctioned violence through decades of systemic divestment and political attack. Drawing on Black feminist, Indigenous, and decolonial theoretical traditions, we propose the *Critical Matriarchal Framework for Health Communication* as a transformative alternative to hierarchical, extractive models of public health practice. This framework centers relational authority, collective responsibility, intergenerational knowledge, and care-based leadership to address and reduce persistent health inequities that are fundamentally rooted in fractured systems of care and community relationships. Using case analyses, authors will demonstrate that without trusted communicators embedded in communities, without frameworks that center reciprocity and care, and without addressing the trauma and structural violence that undermine trust, public health institutions cannot effectively communicate for behavioral changes during emergencies. This intervention has implications beyond public health, offering a model for sectors experiencing similar legitimacy crises and calling for institutional realignment that examines power structures, redistributes leadership, and centers community accountability over bureaucratic metrics. The Critical Matriarchal Framework for Health Communication is necessary because it transforms crisis communication from a technical problem (how to disseminate information) into a relational practice (how to build trust, share power, and co-create solutions with communities). This perspective is about fundamentally reimagining who has authority to speak, whose knowledge counts, and what public health communication is ultimately for: institutional legitimacy and community flourishing. In the context of ongoing and future crises (climate disasters, pandemics, political violence), this framework offers a pathway toward health communication practices that do not just inform behavior but transform systems, that reach communities and honor their wisdom, and that manage crises by building collective capacity for resilience.

## Introduction: the compounding crises in public health

*“What would it be like to drop off my kid at school and then go about my day, knowing someone could drive up to that same building and shoot my baby? People did not matter in this country. I saw it every time I opened the newspaper. Amazon, Open AI, the FBI, ICE, IRS data leaks, Big Oil, book bans, travel bans, bans on gender-affirming care, Do not Say Gay, Dobbs, the Defense lobby, the Finance lobby, the Israel lobby, lead in the water, libertarians, Moms for Liberty, militias…qualified immunity, criminalizing doctors, journalists, protestors, students; the Supreme Court; conspiracies, Citizens United, student debt, school shootings, surveillance, voter suppression, anti-vaxers, evictions, vigilantes, for profit-prisons, predictive policing, patriotic education, private equity, the death penalty, Project 2025, privatized healthcare, the Heritage Foundation, homelessness, harassing librarians, cuts to foreign aid, to Medicare, to medical research, to SNAP, to education, to environmental protection, to epidemic preparedness, to programs for energy efficiency or English Language Learners. I was scared of rising sea levels, toxic waste, wildfires, water shortages, wealth inequality, and war. But that’s not even what scared me most. **What scared me most was me**.”* (39)

### The problem

The contemporary public health disciplinary crisis represents institutional fragility that is the result of state-sanctioned violence. It has been enacted through ongoing systematic divestment, refusal to act on the structural drivers of health inequities, the uplift of western and paternalized knowledge production values and propaganda, and labor exploitation. For the field of health communication, specifically, this has caused a “consequential” shifting of priorities for its fourth decade of institutionalized existence (1). Leading scholars of the field recognize that these multiple crises include the rapid evolution of digital technologies, artificial intelligence, fragmented information environments, global health inequities, declining institutional trust, and intentional misleading information by antagonist actors. The field is, arguably however, slow to recognize the impacts of ongoing, unrelenting, and co-occurring harm on historically marginalized and racialized groups and the necessity for restorative care (2). Culturally responsive and critical health communication scholarship is necessary.

Decades of defunding have hollowed out the infrastructure meant to protect community health, leaving behind a workforce marked by attrition, burnout, and profound demoralization. According to the Council of State and Territorial Epidemiologists, 60% of state health departments report epidemiology staffing shortages, while nearly half of local health departments reduced staff between 2008 and 2019 (3). The COVID-19 pandemic accelerated these losses, exposing the fragility of a system already strained to its breaking point. Experts project that the nation needs an estimated 80,000 additional public health workers to meet basic community health needs. This area of crisis is acutely severe, when understanding that, at its core, public health is a local enterprise.

The workforce collapse must be understood within a broader framework of state-sanctioned violence (4). This is violence that operates through overt acts of harm, calculated neglect, institutional betrayal, and the systematic devaluation of lives deemed expendable. The harm is also emotional and psychological, presenting often as interpersonal, lateral and collective violence. Federal divestment from public health infrastructure is an act of violence. The erosion of institutional credibility through coordinated political attacks is an act of violence. The extraction of expertise from communities of color without reciprocity, acknowledgment, or material support is an act of violence. When the state withdraws resources from the very systems charged with protecting health, it actively produces the conditions for premature death, intergenerational trauma, and community destabilization. These acts have destabilizing downstream impacts that reverberate to the kitchen table, as Black and other marginalized groups seek to safely make sense of their chaotic external and inner worlds (5).

Ecologically, the connection between state divestment and persistent health inequities is structural and intentional. Maternal mortality rates for Black women remain three to four times higher than those for white women (6). Chronic disease disparities persist across generations. Mental health crises intensify without adequate community-based support systems. These disparities persist precisely because public health systems have been designed to operate hierarchically, paternalistically and transactionally, treating health as a service delivered from institutions to individuals rather than as a relational practice rooted in community interdependence and collective accountability (7).

### Our positionality

This commentary is written by three Black women scholar-activists who do not observe this crisis from a distance. We live it. We carry it into institutions that were not designed with our safety, authority, or survival in mind, and we carry it home. The intellectual labor represented in this work is inseparable from the embodied labor of existing and persisting within the very systems we critique. These systems have demanded our expertise while routinely withholding the protections, recognition, and resources that expertise deserves. To write this commentary is itself an act of the praxis it describes. Our analytical framework is grounded in lived experience, in the case examples of women we know and communities we belong to, respect for ancestral intelligence, and in the cultural knowledge and institutional access that our positions have afforded us - forms of knowledge production long sustained within Black matriarchal traditions of care, protection, and collective survival. We name that access not to claim neutrality but to claim its opposite: we write from a specific location, with specific stakes, and we believe that specificity strengthens rather than compromises the scholarship. At the same time, we recognize that the privileges enabling us to publish, convene, and be heard within academic spaces are unevenly distributed; and that the women whose experiences most urgently demand this framework are frequently those with the least institutional protection to speak from. We are accountable to the communities whose knowledge, labor, and survival have informed every argument in this commentary, reflecting matriarchal traditions that understand knowledge as relational, protective, and inseparable from responsibility to community. This work is urgent because the harm is ongoing. We write with that urgency, and with the clarity that comes from knowing, firsthand, what it costs to do this work and what it costs not to.

### State sanctioned violence and public health systems

Public health systems have long operated on an implicit compact of moral commitment and personal sacrifice, demanding that workers absorb risk in service of collective health while systematically failing to ensure their physical, psychological, digital, and reputational safety. This is not an oversight. Decades of federal and state divestment function as a form of institutional violence, one that is distributed, diffused, and deliberately obscured by the language of fiscal constraint and administrative neutrality.

Public health in the United States is experiencing a convergence of crises that cannot be understood as discrete or unrelated failures. Rather, these conditions reflect state-sanctioned violence enacted through policy design, institutional neglect, and communicative practices that expose workers to harm while withholding the protections they are owed. Nowhere is this dynamic more visible than in the experiences of Black women who occupy frontline and leadership roles across public health systems, where the cumulative weight of structural exposure is sharpest and most consequential (8). This commentary advances a Critical Matriarchal Public Health Communication framework to interrogate how violence operates structurally, digitally, and interpersonally within these systems, and how matriarchal logics offer an alternative paradigm organized around care, accountability, and collective survival.

Workforce attrition is most often framed in the literature as a problem of burnout or individual choice, which this commentary explicitly rejects (9). Attrition is more accurately understood as a predictable outcome of unsafe labor conditions, a rational response to chronic exposure to political hostility, public harassment, and institutional abandonment. It is, in this sense, evidence of structural violence that has been normalized as occupational risk. These same conditions produce fragile public health infrastructures by externalizing risk onto individual workers while simultaneously eroding the institutional capacity necessary to respond to threats, misinformation, and targeted harassment. The consequences are not confined to the workforce: they exacerbate the very health inequities that public health systems exist to address and reduce, while destabilizing the workforce tasked with addressing them. Divestment, in this light, is not a neutral policy position; it is a mechanism that redistributes harm downward.

This commentary defines state-sanctioned violence as harm that is enabled through policy gaps, maintained through institutional inaction, and legitimated through political rhetoric, lateral violence in interpersonal relationships, and strategic silence. Within this framework, the harassment, doxxing, and stalking of public health workers are not aberrations to be addressed through individual resilience or procedural remediation. They are foreseeable outcomes of intentional governance choices that have consistently prioritized political expediency over worker protection. The following illustrative case demonstrates how these dynamics unfold in practice, illustrating how structural divestment, communicative delegitimation, and institutional inaction converge to produce environments in which violence against public health workers becomes normalized, while matriarchal frameworks illuminate alternative logics of protection, accountability, and collective care.

#### Illustrative case: institutional exposure and compounded harm

In this case, a public health professional engaged in community-based advocacy and public communication became the target of sustained online harassment following routine professional activities (10). The harassment included misrepresentation of professional conduct, persistent digital defamation, and the circulation of personal information across platforms. Institutional responses were fragmented and largely procedural, emphasizing individual documentation and self-management rather than coordinated protective action. Despite clear escalation over time, the absence of enforceable safeguards allowed digital aggression to persist and migrate beyond online spaces.

As the harassment intensified, it intersected with interpersonal threats that required navigation across multiple systems, including employment structures, legal processes, and personal safety planning. Notably, the burden of proof, risk mitigation, and reputational repair rested almost entirely on the individual, reflecting broader patriarchal policy designs that presume neutrality while producing gendered and racialized harm. This case underscores how political delegitimation of public health expertise, combined with institutional divestment from worker protection, creates conditions in which harassment is normalized and safety is privatized. The resulting suppression of professional voice, intellectual contribution, and public engagement illustrates how violence against public health workers functions not only as individual harm, but as a mechanism that constrains knowledge production and undermines health equity efforts at the population level.

### The particular vulnerability of black women

The departure of over 300,000 Black women (11) from the workforce since the pandemic is a deliberate outcome of systems designed to extract care labor while denying the resources, recognition, and safety necessary to sustain it. For these public health professionals, daily work has become an exercise in navigating grief, fear, and confusion alongside unwavering dedication to communities under siege. Within a broken public health system, their labor is consumed by managing crisis after crisis — treating symptoms rather than causes and responding to widening inequities rather than preventing them. Their expertise is routinely dismissed, their leadership potential constrained, and their well-being sacrificed to institutional preservation. The intersection of racial, gender, and professional marginalization creates conditions where Black women bear disproportionate emotional, physical, and intellectual burdens while receiving the least institutional support, advancement opportunities, or protection from harm.

Their departure signals collective abandonment by institutions that rely on their labor yet refuse to validate their expertise, protect their well-being, or invest in their professional growth. The loss of these practitioners destabilizes not only public health organizations but entire care networks and community trust systems. When Black women leave the field, they take with them decades of community relationships, cultural humility, translation capacity, and the lived knowledge that bridges institutional systems and the communities they purport to serve. This represents a compounding crisis: workforce attrition simultaneously reflects and reproduces the very health inequities public health exists to address. This commentary centers the knowledge traditions developed through Black women’s collective experiences at the intersection of racialized labor expectations, gendered norms of care and resilience, and the devaluation of public health expertise. These forces do not operate independently; instead, they converge and compound, magnifying exposure to violence while simultaneously constraining the avenues through which affected workers might seek redress or relief. To occupy this intersection means absorbing disproportionate institutional demands while receiving little institutional protection. Within a crisis framework, this dynamic that renders harm more likely and more difficult to name, confront, or remedy.

### Scope and purpose of commentary

This commentary contributes to the special issue by positioning public health workforce collapse as a crisis of legitimacy rooted in structural violence. We argue that rebuilding public health demands fundamental transformation of leadership models, evidence hierarchies, and communication practices. Specifically, we propose Critical Matriarchal Futures as a framework for reimagining public health communication leadership that resists rather than replicates violence (12). Matriarchal frameworks matter now because, as institutions face compounding pressures, the temptation toward authoritarian crisis management intensifies. Critical matriarchal approaches offer an alternative pathway grounded in tested governance principles from Black feminist, Indigenous, and decolonial traditions. These principles prioritize relational authority, intergenerational knowledge, and community-rooted accountability over command-and-control structures.

This piece proceeds in four movements. First, we establish the theoretical foundations of Critical Matriarchal Futures and articulate why matriarchal frameworks are particularly suited to public health communication challenges. Second, we analyze how conventional public health communication reproduces rather than disrupts state-sanctioned violence through evidence hierarchies, expert gatekeeping, and community erasure. Third, we present concrete applications of matriarchal communication principles through case studies and emerging models. Finally, we outline implications for practice, policy, and institutional transformation, acknowledging both the radical potential and material constraints of this reframing.

## Theoretical framework: critical matriarchy and public health communication

### Defining critical matriarchal futures

Critical Matriarchal Futures is an analytic framework that centers relational accountability, collective survival, and community protection as guiding principles for governance, knowledge production, and public health communication. Rather than positioning matriarchy as the inverse of patriarchy, the framework examines how traditions of relational leadership and caregiving knowledge, long practiced within Black, Indigenous, and other colonized societies, offer alternative logics for organizing institutions and responding to structural violence (13, 14). The theoretical genealogy of Critical Matriarchal Futures emerges from multiple intellectual traditions that have long contested Western patriarchal knowledge systems. Black feminist scholars, including Patricia Hill Collins, bell hooks, and the Combahee River Collective established foundational critiques of interlocking oppressions and articulated alternative epistemologies rooted in community accountability and collective struggle (15, 38, 40). Indigenous scholars such as Kim TallBear, Eve Tuck, and Leanne Betasamosake Simpson have theorized governance systems organized around kinship relations, ecological reciprocity, and multi-generational responsibility (45-47). Decolonial feminists, including María Lugones, Sylvia Marcos, and Oyèrónkẹ́ Oyěwùmí, have demonstrated how colonial violence imposed patriarchal gender systems onto societies with fundamentally different organizational logics, making the recovery and reimagining of pre-colonial structures an act of epistemic resistance (42-44).

In this commentary, we apply Critical Matriarchal Futures specifically to the domain of public health communication, examining how matriarchal governance principles reshape the production, circulation, and protection of public health knowledge. Critical Matriarchal Futures distinguishes itself from both patriarchal and liberal feminist approaches through its ontological commitments. The framework does not advocate the replacement of patriarchal authority with gendered reversal or biological essentialism; rather, it examines relational, collective, and anti-extractive modes of governance historically sustained within marginalized communities. In patriarchal systems, authority is concentrated in individual male leadership and treat care as the responsibility of subordinated others. In contrast, matriarchal frameworks distribute leadership across kinship and community networks and position caregiving knowledge as strategic expertise. Where liberal feminist approaches seek women’s inclusion within existing hierarchical structures and individual advancement within competitive markets, Critical Matriarchal Futures questions the legitimacy of hierarchy itself. It advocates for collective liberation through structural transformation. The use of the term *matriarchal* is deliberate, as is the use of *critical*. It signals the inclusion of women within existing institutions, the centering and uplifting of the protection of children, and the reorganization of authority around relational responsibility, community protection, and collective stewardship.

Within health communication, this includes “work that bridges theory and practice, spans local, national, and importantly global contexts, and interrogates the social, institutional, and technological infrastructures that increasingly define” our field [(1), p.1]. Scholars have long called for a *critical* lens which includes an “agenda of interrupting the theory and practice of health communication in the mainstream, bringing to the forefront questions of social justice, equity, participation, and structural transformation” [(16), p.534]. This commentary also raises questions about the historical valuation of knowledge production within health communication scholarship. Scholars working from marginalized and racialized positions have long examined the epistemic hierarchies that shape whose knowledge is recognized as authoritative within the field. Recognizing this history matters because inclusion within violent institutional structures does not, by itself, constitute justice. The presence of women, or other previously excluded groups, within hierarchical institutions may represent symbolic progress, yet such representation does not necessarily transform the underlying logics of domination and extraction that organize those institutions (17). Critical Matriarchal Futures therefore shifts the central question from “how do we get more representation in leadership?” to “what would leadership look like if organized around care, reciprocity, and collective accountability rather than domination and extraction?”

### Why matriarchal frameworks for public health communication?

Public health communication is particularly well-suited to transformation through matriarchal frameworks because health itself is fundamentally relational and structural. Health emerges from individual choices or clinical interventions primarily and also from the quality of relationships, the integrity of care networks, the distribution of resources, and the conditions that enable or constrain human flourishing across generations. Ignoring this distribution of responsibility is harmful. Yet, conventional public health communication often treats individuals as isolated decision-makers who need better information to make optimal choices, ignoring the relational contexts that shape both health and the capacity to act on health-promoting knowledge and the co-occurring crises that exist (2).

Critical approaches to health also situate it as a human right which requires examining, globally, whether communities possess the material conditions, social support, cultural integrity, and political power necessary for collective well-being (18). Matriarchal frameworks operationalize health as a human right by centering questions of distribution, reciprocity, and collective responsibility: Who bears the costs of health-producing labor? Whose knowledge counts in defining health problems and solutions? How are resources allocated across communities and generations? What relationships enable or constrain communities’ capacity to protect their members’ health?

Medical-institutional models of public health communication have reached their limits (19, 20). Despite decades of health education campaigns, health literacy initiatives, and evidence-based messaging, persistent inequities remain or worsen (21). These approaches fail because they: (1) misunderstand the problem; and (2) misdirect the messaging elucidating the structural drivers of the problem. Treating health disparities as information deficits ignores how structural violence, historical trauma, institutional racism, and community divestment shape both health outcomes and trust in institutions. Communities experiencing medical apartheid, social services access inequalities, environmental racism, and intergenerational poverty do not lack knowledge about healthy behaviors; they lack the resources, support systems, and institutional trustworthiness necessary to enact that knowledge under hostile conditions. Critical matriarchy approaches are desperately needed epistemically within the health communication discipline.

Contemporary organizational research further validates matriarchal approaches, demonstrating that organizations led by principles of distributed leadership, psychological safety, and care-centered decision-making exhibit greater resilience during disruption and transformation. During the COVID-19 pandemic, communities and organizations that relied on mutual aid networks, horizontal coordination, and collective problem-solving often responded more effectively than hierarchical institutions paralyzed by risk aversion or hampered by rigid chains of command (22). Matriarchal frameworks build adaptive capacity by distributing knowledge across networks, normalizing flexibility and experimentation, and treating care for workers as essential infrastructure rather than luxury to be sacrificed under pressure. The framework does not advocate the replacement of patriarchal authority with gendered reversal or biological essentialism; rather, it examines relational, collective, and anti-extractive modes of governance historically sustained within marginalized communities.

### Critical matriarchal framework for health communication as intervention

Urgently applying critical matriarchal frameworks to public health communication can transform narratives around trust, efficiency, prevention, crisis response, restorative care, and community connectedness. Evidence-based intervention has long served as the cornerstone of public health practice, guiding decisions and shaping outcomes across populations. Yet the conventions through which evidence is produced, leadership is structured, and care is delivered have themselves become sites of inequity. This commentary proposes a Critical Matriarchal Framework for Health Communication as Intervention. We reimagine what constitutes evidence and expertise, who holds authority, and how systems of care are built and sustained. This framework rests on three essential and interrelated components: reimagining evidence and knowledge production, restructuring leadership and accountability, and shifting from service delivery to genuine systems of care.

#### Reimagining evidence and knowledge production

*“Imagination is one of the most powerful modes of resistance that oppressed and exploited folks can do and use.”* (23)

Dominant approaches to evidence production in public health have relied heavily on institutional and administrative data, treating population-level metrics as the authoritative standard while systematically overlooking and co-opting community-derived knowledge (17). This hierarchy is rooted in epistemological traditions that have historically dismissed the expressed needs, lived experiences, and interpretive insight of communities, and particularly communities of color, as insufficiently rigorous or scientifically valid. The Critical Matriarchal Framework for Health Communication challenges this hierarchy directly. Rather than locating expertise exclusively within institutions, this framework centers community-driven evidence as both methodologically legitimate and strategically necessary. The shift is participatory rather than authoritative, and it carries material consequences: when communities are invested as co-producers of knowledge, interventions become more sustainable, more contextually grounded, and more likely to address the conditions that data alone cannot fully capture. This includes ancestral intelligence and community-based knowledge systems that have too often been rendered invisible by conventional research design.

Central to this reimagining is the principle of data sovereignty, meaning the rights, control, and power over data transferred to the community level (24), where members have access to, ownership of, and the capacity to use data to make informed decisions for and by themselves. Caregiving knowledge, often feminized and thus devalued in professional hierarchies, is repositioned here as strategic expertise. Methods such as photovoice (25), daughtering (26), and other community-based participatory approaches offer concrete mechanisms for integrating community interpretation into evidence production, challenging the overrepresentation of statistical significance as the default standard for knowledge and expanding what counts as valid input (27) during needs assessments, planning, and implementation.

#### Restructuring leadership and accountability

*“The relationship between the establishment and what we do as activists is complicated by the inherent tension that is the establishment that we are both dependent upon and fighting against.”* [(28), p. 238]

The framework’s second component requires a fundamental rethinking of how public health leadership is structured and to whom it is accountable. Traditional models of public health leadership vest authority in individual actors operating through formal institutional channels. This is a historical arrangement that prioritizes hierarchical control and paternalized frames over relational effectiveness. The Critical Matriarchal Framework for Health Communication instead advocates for leadership distributed across community kinship networks, recognizing that social ties (both direct and indirect) are among the most powerful determinants of behavioral influence, trust, and sustained change.

Under this model, leadership is not transactional in the narrow sense but relational and network-oriented, emphasizing long-term community well-being over short-term metrics that often serve performative or bureaucratic purposes rather than genuine community welfare. When accountability shifts from institutional bureaucracy to community-driven structures, transparency deepens and trust becomes possible in ways that top-down governance rarely achieves. Grassroots organizing efforts, such as Family listening/Family Circle Program in New Mexico, Lucinda’s House in Hartford, Connecticut, SisterSong in Atlanta, Georgia, the Amos Project in Cincinnati, Ohio and Moses in Detroit, Michigan, offer instructive examples of what becomes possible when leadership prioritizes relationships and accountability flows toward the community rather than upward through administrative channels. These cases, along with models drawn from Indigenous organizing traditions, demonstrate that community-rooted leadership produces interventions that are more effective, endear community trust, and sustainable beyond short-term or inconsistent funding priorities.

#### From service delivery to systems of care

*“As organizations begin to recognize the impacts of colonization on their leadership structures and the broader health system, a powerful act of reconciliation and decolonization is supporting the reawakening of feminine structures in organizations.”* Defriend & Cook (29)

The third and integrating component of this framework calls for a structural reorientation of intervention itself. Interventions, traditionally in western society, have a connotation that positions health as an institutional service delivered to individuals (30). An intervention based on critical matriarchal tenets moves toward a comprehensive, communal system of care. Service delivery models are extractive by design: they collect information, measure outcomes, lean towards capitalistic gain, and produce data that rarely returns meaningful benefit to the communities from which it was drawn. Systems of care, by contrast, are reciprocal. They are built on trust, sustained through ongoing relationships, prioritize restorative repair, and anchored in hyperlocal structures that engage community members as researchers, health ambassadors, and co-designers of their own care environments.

Community health workers (CHW) exemplify this model in practice. As trusted liaisons between communities and formal health systems, they navigate institutional complexity on behalf of residents, provide culturally grounded health education, and advocate directly as embedded members of the communities they serve (31). Austin and Qu (32) champion strongly that CHWs are health care organizations’ frontline workers who extend promotive, preventive, and curative health care services in the communities they serve. Substantial scholarship validates CHWs role in communal care: advocating for individual health needs, implementing programs that educate clients, providing in-home care as necessary, promoting awareness about social welfare issues, such as domestic violence or alcohol and drug abuse, and providing preventive services such as blood pressure, glaucoma, and hearing screenings (33).

#### Addressing persistent inequities through a relational Lens

*“…the violence and oppression endured through [institutions] are often channelled against each other, wherein community, relatives, and family systems continue to treat each other with competition or disrespect.” Defriend & Cook* (29)

Taken together, these three components offer a framework for addressing the persistent health inequities that conventional intervention models have failed to resolve. Maternal mortality, chronic disease disparities, collective trauma, and environmental injustice are not reducible to individual behavioral deficits or gaps in service access. They are relational crises and products of fractured community ties, intergenerational harm, and systems of care that were never designed with equity as their organizing principle. Shifting how we communicate these realities is an urgent imperative. The Critical Matriarchal Framework for Health Communication insists that interventions adequate to these crises must be built from the same relational logic that sustains communities under conditions of structural neglect: care as practice, accountability as relationship, and knowledge as something held collectively rather than extracted and owned.

Hanisch (34) coined the term ‘the personal is political,’ which speaks to an important element of this framework. While this commentary focuses on structural and institutional accountability, it is imperative that accountability also rest on how individuals choose to exist in community — whether as neighbors, workers, family members, or parents. Each of these roles is a site of political choice, and each offers an opportunity to either reproduce or refuse the logics of harm that structural analysis helps us name. The personal and the collective are not in tension here; they are inseparable. To work toward health equity is to commit, simultaneously, to transforming systems and to transforming ourselves. This demands that community members develop the critical tools to interrogate, erotically, the narratives that surround them, including those delivered through media (35).

The framework also calls for a decisive reorientation away from the cultural logics of individualism and toward the sustained practice of collective care. It requires resisting the privatization of suffering and embracing instead a shared responsibility for the conditions that make flourishing possible or impossible. This means showing up in the ordinary and ongoing work of community maintenance. When harm does occur, the framework insists on restorative rather than punitive responses. Restorative practices center the humanity of everyone involved, creating structured space for acknowledgment, accountability, and repair. They recognize that conflict, when met with honesty and care, can be a site of deepened relationship rather than permanent rupture. Conflict resolution, in this context, is not the suppression of disagreement but the practice of moving through it without abandoning one another. It is rooted in the belief that communities are strengthened, not weakened, when they develop the capacity to face hard truths together. Underlying all of these commitments is an insistence on moving with love as orientation (36). Love, in this framework, is the disciplined refusal to treat people as disposable, the willingness to remain in accountability, and the insistence on dignity even under conditions designed to erode it.

### Implications for health equity praxis

A framework is only as consequential as the conditions it creates for transformation. This commentary would not be sufficient if there was not an intentional linkage to how this work will continue to campaign towards health equity. Communication gaps are cited as a key barrier to healthcare access and to achieving health equity (37). The Critical Matriarchal Framework for Health Communication is a call to praxis, demanding that institutions, practitioners, scholars, and communities engage in the difficult, sustained, and often uncomfortable work of structural realignment. This section outlines what that work requires across institutional, organizational, methodological, and cross-sectoral dimensions, while naming honestly the resistance, barriers, and measurement challenges that any serious equity-oriented framework will inevitably encounter.

#### Institutional realignment: doing the hard work

For institutions, adopting this framework requires a genuine examination of power. For example, who holds it, how it was accumulated, and whose labor has historically subsidized it. Public health institutions that operate within the logic of extraction, whether extracting data, labor, or legitimacy from communities without reciprocal investment, cannot become systems of care simply by rebranding existing practices. The hard work of institutional realignment means confronting the organizational cultures, hiring practices, funding priorities, and accountability structures that have consistently redistributed risk downward while concentrating resources and upward recognition.

At the policy and practice level, this demands concrete changes: formal protections for public health workers against harassment and political interference; equitable compensation structures that reflect the full scope of expertise (including democratizing the value of community and caregiving knowledge) that practitioners bring to their roles; and governance mechanisms that make community accountability structurally legible rather than aspirationally rhetorical. It also requires that institutions examine their internal power structures with the same analytical rigor they apply to external health disparities. Institutions that have derived legitimacy from hierarchical arrangements of expertise and co-opting of marginalized voices and peers will not voluntarily relinquish those arrangements without sustained pressure from workers, communities, and the scholarly literature that names their costs.

#### Scaling and dissemination considerations

The question of how a matriarchal framework travels across institutional contexts is both logistical and political. In academic public health programs, dissemination begins with curriculum. Knowledge that is accessible requires that students are trained to recognize community-driven knowledge as methodologically rigorous, whether intersectional analysis is treated as foundational rather than supplementary, and whether future practitioners are prepared to work within systems of care rather than systems of service. Syllabi, training models, and accreditation standards are sites where the boundaries of legitimate knowledge are drawn and enforced, and they are therefore sites where this framework must be introduced and defended.

State and local health departments and government agencies pose different challenges. Public institutions operate within political environments that can shift rapidly, as recent years have made painfully clear, and the workers most committed to equity-centered practice are often the most vulnerable to that volatility. Scaling this framework in government settings requires institutional policies that protect equity-oriented work from political cycles. For community-based organizations, many of which already practice matriarchal logics without naming them as such, the challenge is less about adoption than about recognition. There must be a commitment to ensuring that the intellectual and relational labor of grassroots organizations is credited, resourced, and protected rather than absorbed into academic or institutional frameworks that will claim the insights without sharing the credit. Potential barriers to adoption across all these settings include funding structures that incentivize compliance over transformation, leadership cultures resistant to distributed accountability, and the persistent devaluation of the women (particularly Black women) most likely to be doing this work.

#### Measurement and evaluation challenges

Perhaps nowhere is the tension between conventional public health practice and a matriarchal framework more acute than in measurement. Traditional evaluation models are designed to produce outcomes legible to funders: quantifiable, time-bounded, and amenable to comparison across populations. While these metrics do not have value, they are insufficient and, in some cases, actively distorting when applied to frameworks whose core commitments are relational, long-term, and community-defined. Rarely is measurement also interrogated against a critical lens.

What counts as success within a matriarchal framework cannot be reducible to a dashboard. Trust built over years between a community health worker and the families she serves is not captured in a six-month outcomes report. The restoration of community efficacy following decades of institutional abandonment cannot be resolved within a grant cycle. We must uplift and validate alternative metrics, such as measures of relational depth, community self-determination, narrative shift, and harm reduction, that never make it into administrative data When accountability flows primarily to funders rather than to communities, evaluation becomes a mechanism of institutional self-protection rather than a genuine instrument of learning and improvement. The framework insists on reversing that orientation, even when doing so creates friction with the financial architectures on which public health work depends.

#### Beyond public health: broader applications

The conditions that have produced co-occurring and never-ending crises in public health are not unique to this sector. Education, social work, humanities, and community organizing are experiencing parallel forms of workforce attrition, driven by the same structural logic: systems that demand moral commitment, deference to new technologies in knowledge production, and personal sacrifice from workers while failing to provide safety, recognition, empathy, or adequate compensation. In each of these fields, the practitioners most exposed to harm are also those most likely to be women of color, most likely to be doing the relational and caregiving labor that holds institutions together, and least likely to be protected when those institutions come under political or fiscal pressure.

The Critical Matriarchal Framework for Health Communication, therefore, has applications beyond public health, offering a transferable logic for any sector grappling with the convergence of workforce crisis, equity failure, and institutional legitimacy. Taking the aforementioned framework seriously across various sectors means recognizing that the public health crisis is not an anomaly but a symptom of deeper issues. The conditions required for achieving genuine equity praxis are the same, whether one is designing a maternal health intervention, building a school, or organizing a neighborhood. These conditions include viewing care as essential infrastructure, fostering accountability as relational processes, and valuing women’s labor as vital–not as a disposable or easily co-optable resource–but as the foundation upon which any system for collective survival must be built.

## Conclusion: toward transformative futures

*“The question I ask is ‘[w]ill the interregnum, the crisis, whose historically normal solution is blocked in this way, necessarily be resolved in favour of a restoration of the old?’* (41)

The conditions documented throughout this commentary are not problems that incremental reform can resolve. They are symptoms of a paradigm in crisis — one that has exhausted its capacity to sustain the workers, communities, and relationships upon which public health depends. What is required is a fundamental reorientation of its premises: who counts as an expert, who bears risk, who holds power, and to whom public health systems are ultimately accountable. The Critical Matriarchal Framework for Health Communication advanced in this commentary draws on the forms of knowledge, leadership, and care that communities have practiced for generations under conditions of structural neglect. This is fitting, because public health is, at its core, a relational discipline and health communication is its lynchpin. The task, then, is to recognize, resource, and protect these community-rooted practices. And where institutional silence has functioned as complicity in harm, this framework demands that such silence be rejected.

### Tensions and contradictions

Intellectual honesty requires naming the contradictions that this framework must navigate rather than resolve. To advance a matriarchal framework within academic publishing is already to operate inside the institutional structures this commentary critiques. The funding that supports public health research and intervention often flows from the same foundations, agencies, and government bodies whose policy choices have produced the conditions of harm described here. Professionalization threatens to minimize and invalidate the contributions of grassroots frameworks, translating community-rooted logics into institutional language until what remains is palatable but no longer transformative. And there is a real and persistent tension in the production of academic knowledge about community knowledge. This is a tension that this commentary has not fully escaped, and that future scholarship in this tradition must continue to examine with rigor and humility. These contradictions name the terrain on which it must operate. Transformation rarely happens from outside systems entirely; it happens at the friction points, where those committed to change work within structures they are simultaneously working to dismantle.

### Engaging potential critiques

This commentary anticipates several substantive critiques that deserve direct engagement. The first concerns essentialism: the risk that a matriarchal framing inadvertently naturalizes caregiving as the domain of women, reproducing the gendered logic it seeks to challenge. Matriarchal logics are not rooted in biological determinism but in the social, political, and epistemological traditions of communities where women have historically held relational and organizational authority under conditions that formal institutions refused to recognize. The framework honors that history without prescribing gender.

Relatedly, this commentary affirms the full inclusion of non-binary, gender-expansive, and trans persons within a matriarchal framework. Care, relational accountability, and community-rooted knowledge are not the exclusive province of cisgender women, and any application of this framework that forecloses gender expansiveness has misunderstood its foundations. The framework also carries a responsibility to avoid romanticizing or appropriating Indigenous practices and knowledge systems. Where Indigenous organizing, governance traditions, and epistemologies have informed this work, that debt must be named, credited, and accompanied by concrete commitments to Indigenous sovereignty and self-determination, and not absorbed into academic frameworks as evidence without attribution or reciprocity. Finally, the risks of scalability and institutionalization are real: frameworks that begin in resistance frequently become instruments of the institutions they were designed to challenge. Vigilance against that absorption is not a one-time commitment but an ongoing practice.

### A call to action

For public health practitioners, this framework is an invitation to refuse the normalization of harm as occupational risk. It is the work that so many historically marginalized and racialized folks must also do in their personal lives - to name state-sanctioned violence as such, to document it, to organize around it, develop health interventions that value it, and to insist that no public health mission is worth the systematic sacrifice of the workers who carry it. For academic institutions, the call is to examine whether curricula, promotion structures, publication hierarchies, and research partnerships reproduce the same epistemic and material inequities that public health claims to address and to make the institutional changes, not merely the rhetorical ones, that genuine accountability requires. For communities, this framework affirms what many already know: that the knowledge, leadership, and care that have sustained collective survival under conditions of structural abandonment are not deficits to be supplemented by institutional intervention but assets to be protected, resourced, and centered. For policymakers, the demand is to enact formal protections for public health workers, invest in the workforce and the communities it serves, and accept that governance choices carry consequences for which accountability cannot be indefinitely deferred.

### Future directions

This commentary opens as many questions as it resolves, and that is by design. Future research emerging from the framework should examine how matriarchal logics operate across different regional, cultural, and institutional contexts; how data sovereignty initiatives can be structured to protect community ownership against institutional appropriation; and what the longitudinal outcomes of distributed, kinship-networked leadership look like when measured on community-defined terms. Policy work must pursue formal recognition of community-based expertise in funding eligibility, workforce classification, and evidence standards. These changes will require both legislative strategy and sustained pressure from the public. Movement-building strategies should prioritize protecting and amplifying Black women already doing this work, ensuring that the framework’s scholarly articulation translates into material support rather than additional extraction.

The vision this commentary holds is a public health system that does not abandon its own. It is bold to imagine a disciplinary and practice system that treats the safety of its workers as inseparable from the safety of the communities they serve, that recognizes care as the infrastructure upon which all other public health work depends, and that understands the labor of Black women not as a resource to be consumed in crisis but as the generative center of any system worthy of the name. That vision is not beyond reach. It is already being practiced, in fragments and in fullness, in the communities, clinics, organizing tables, and kitchen tables where people have always done the work that institutions have not. The task now is to build systems that are finally adequate to them.

### Limitations

While centering women’s experiences in the proposed matriarchal framework, we acknowledge its limitations. First, the proposed matriarchal framework approach might inadvertently risk masking non-binary identities by focusing solely on gender as binary - female and male. For example, in the context of our proposed framework to produce knowledge, data collection, policy development, or narrative framing with only a women-led experience rather than gender neutrality might be overlooked, which is not the intention, but is a structural limitation that impacts knowledge production. Second, a matriarchal framework can unintentionally reproduce harmful stereotypes, such as those around caregiving and moral virtue, which are less violent than patriarchal frameworks but still need to be inclusive of social norms that do not perpetuate harmful social norms or maintain the status quo. Lastly, Critical Matriarchal Futures therefore seeks not to romanticize (Black) women’s labor or invert patriarchal power structures, but to interrogate and reimagine the underlying institutional logics that normalize domination, extraction, and disposability. Addressing these tensions is essential to strengthening the framework’s inclusivity, analytical rigor, and applicability across diverse experiences of structural violence.

## Data Availability

The original contributions presented in the study are included in the article/supplementary material, further inquiries can be directed to the corresponding author.
